# Feeding Corn-smut to Dairy-cows1Michigan State Agricultural College Experiment Station, Bulletin No. 137.

**Published:** 1898-02

**Authors:** Clinton D. Smith

**Affiliations:** Director of the Michigan Agricultural Experiment Station


### FEEDING CORN-SMUT TO DAIRY-COWS.[1]

By Clinton D. Smith,
DIRECTOR OF THE MICHIGAN AGRICULTURAL EXPERIMENT STATION.

Professor C. F. Wheeler, the botanist of the station, reports as follows concerning the life-history of corn-smut and the precautions to be observed to avoid its further extension:

"*Life-history.* In an address delivered before the Agricultural Club, at Berlin, Germany, February 11, 1888, Dr. Oscar Brefeld, first gave to the world the true life-history of corn-smut. For twelve years he had experimented with the greatest care, both in the field and in the laboratory, to learn the actual behavior of the smuts of cereals.

"It had been proven before this time that the smuts of wheat, barley, and oats enter the sprouting grain when the young stem is less than one-quarter of an inch in length, and grow upward with

[1] Michigan State Agricultural College Experiment Station, Bulletin No. 137.

the growth of the stem until the grains in the head are formed, when they at once seize upon this storehouse of prepared food, appropriating it to their own use. Until 1887 it was supposed that corn-smut followed the same course of development.

"The crowning glory of Brefeld's discovery is in proving that corn-smut may infect the plants at any time before their full maturity, and, moreover, that the ripened spores themselves, by falling upon the corn-plants, do not directly produce the disease.

"These black, dusty masses of ripened smut-boils contain multitudes of dark-brown spores well protected with thick walls, making them capable of resisting extremes of temperature and moisture and retaining their germinating power for a number of years.

"These spores were found by Brefeld to germinate readily outside and away from corn-plants whenever placed in moist, fresh manure. He also found that they would not germinate readily in pure water, but that in manure-water the spores germinate rapidly, forming a multitude of short branches which produce secondary spores in great numbers. These secondary spores are formed in the air, on the surface of the water, also beneath it and are easily carried by winds to fields, falling upon all parts of corn-plants. Dew and rain carry these small spores inside the leaf-sheaths and the husks of the ears, where they at once send out germ-tubes that enter the plants, producing a local disease (the well-known smut-boils) near the place of infection. In two or three weeks after the infection the characteristic smut-boils appear.

"This disease has been known since the discovery of America, and has followed the introduction of corn-raising into all parts of the world.

"The loss to the corn-crop is rarely a serious one, in ordinary years not averaging over 2 per cent.

"*Prevention.* No remedy for corn-smut is known. From the above account of its life-history, it is evident that it is useless to treat the seed previous to planting. No doubt fungicides sprayed upon the growing corn might check the disease, but this treatment is not practicable.

"Certain precautions may lessen the disease, for instance, cutting out and destroying smutted parts before the spores ripen, and the use of chemical fertilizers and well-rotted manure, with a reasonable rotation of crops. The use of manure from stock fed with silage of smutted corn certainly tends to increase the liability to infection in fields to which such manure is applied. Our knowledge of the manner in which the disease is carried from field to field is wanting."

The unusual prevalence of smut in the College cornfields in the fall of 1895 furnished the material for an experiment to test the effect of feeding corn-smut both in moderate and excessive quantities to dairy-cows.

The smut was gathered after the corn was cut, and no attempt was made to entirely separate the husks and small abortive ears from the actual smut-boils. Many masses of smut fell to the ground and brought away with them small quantities of sand. The figures given in the tables below, therefore, indicate not the exact amount of smut consumed, since with the smut there was a small but uncertain per cent. of sand, husks, small pieces of leaves, and abortive ears. The smut was drawn to the barn as fast as gathered and stored in bulk. It suffered no apparent fermentation or change of any kind before it was fed.

The smut was given to the cows mixed with their grain-ration, which consisted of corn, four parts; wheat-bran, three parts; ground-oats, two parts, and oil-meal, one part. The rough feed consisted of cornstalks cut into inch lengths and a small ration of hay, never exceeding six pounds per day, thus compelling the cows to derive the principal part of their nourishment from the cornstalks, the smut, and the grain-ration.

The four cows used in the experiment were grades, apparently vigorous and healthy, purchased in the vicinity of the College. The essential facts in regard to them are as follows:

| Name. | Breed. | Age, years. | Due to calve. |
|---|---|---|---|
| Materna . . . | Shorthorn . . . | 2 . . . | February 5, 1896. |
| Milla . . . . | " . . . | 7 . . . | December 5, 1895. |
| Hebe . . . . | " . . . | 5 (?) . . | February 15, 1896. |
| Halo . . . . | Jersey . . . . | 7 . . . | August 1, 1895. |

The experiment began November 6, 1895.

Materna and Milla, the former six months in calf at the beginning of the experiment, and the latter due to calve early in its progress, were fed corn-smut in as large doses as they could be induced to receive it.

Halo, fresh in milk, and Hebe, six months in calf at the beginning of the experiment, received the smut in moderate doses only.

To each of the two cows, Hebe and Halo, two ounces of smut were given daily from the 7th to the 12th of November. On the 12th this quantity was doubled, each cow receiving four ounces.

On the 13th the ration of smut was again doubled, and each cow was given one-half pound. This quantity was again increased on the 15th to twelve ounces, and, finally, on the 17th, to one pound per day. A pound of smut in the condition existing at the time of the experiment would fill a two-quart measure.

For Materna and Milla, on the other hand, the dose was very rapidly increased from two ounces per day on the 7th and 8th of November, to four ounces on the 9th and 10th, six ounces on the 11th and 12th, twelve ounces on the 13th and 14th, one pound and a quarter on the 15th and 16th, one pound and three-quarters on the 17th and 18th, and finally two pounds per day of the smut each, from the 19th of November to the 12th of December, inclusive. In order to test the matter fully the amount given these cows was very rapidly increased after the 13th of December. On that day they each received three pounds of smut, on the 14th four pounds, on the 15th five pounds, on the 16th six pounds, on the 17th seven pounds, on the 18th ten pounds, and finally, on the 19th, eleven pounds, when the experiment closed. Ten pounds of smut shoveled into a half-bushel measure filled it. It is evident that the cows received in this daily ration more smut than they could possibly get in foraging over a cornfield after the removal of the crop, or in the stables in the winter when fed exclusively on cornstalks as the roughage of the ration.

At the beginning of the experiment the cows ate the smut with great avidity, and the two cows, Hebe and Halo, who received it in moderate quantities only, continued to prefer it to their grain-feed up to the close of the experiment. The two cows who received it in immoderately large quantities, on the other hand, manifested a less liking for it as the quantity was increased, although they did not reject it up to the very last day of the experiment.

No change in appearance was noticed in the dung until the 22d of November, when it was observed to be distinctly darker than that of other cows in the stable fed a similar ration of grain and fodder without the smut. In consistency it was somewhat harder than normal, and possibly in the cases of Materna and Milla somewhat scantier.

Except in the case of Milla, who dropped her calf on the 5th of December, the weights of the cows for the most part gradually increased. Certainly no ill-effect was noticed which could be ascribed to the feeding of corn-smut.

An examination revealed no variations in temperature that could be ascribed to the corn-smut. Low temperature of Materna on November 8th was due to her having been exposed to a cold rainstorm for a couple of hours previous to the taking of the temperature. The temperature of Milla dropped suddenly just prior to her calving.

The pregnant cows were watched for signs of abortion, but none appeared.

Their milk-yield was regular and constant, in the case of the cows giving milk, and no indication was given of any variation in this respect from normal conditions.

On the very last day of the experiment five pounds of the corn-smut were given in the morning and six pounds at night to Materna and Milla. The following morning it was found that the night feed had not been entirely eaten up, approximately three-quarters of a pound of the mixed grain and smut being left in each manger. Their appetites for smut seemed to have been completely satisfied for the first time during the experiment.

On December 20th four pounds of smut were again fed these cows, mixed as usual with their grain-feed. While the cows did not absolutely refuse the mixture, they ate it with very evident reluctance. The bowels of Milla were loose from the heavy feeding of the day previous, and the cow seemed decidedly indisposed. Her temperature rose to 103° on the evening of December 19th, the day on which she received the eleven pounds of smut.

The behavior of the two cows, Materna and Milla, was watched for a week thereafter. They continued in good health, and gave no signs of any abnormal condition of the bowels. Their dung gradually lost the dark color, and the cows then became in every way normal.

Samples of the corn-smut were placed in the hands of the chemist for analysis, with the following results: Moisture, 8.30 per cent.; albuminoids, 13.06 per cent.; carbohydrates, 25.60 per cent.; cellulose, 24.69 per cent.; sugar, 4 per cent.; fat, 1.35 per cent.; ash—much sand, 22.50 per cent.

Dr. R. C. Kedzie, the chemist of the station, in commenting on this analysis, says:

The ash like that of grain was rich in the phosphates of potash and magnesium, but a large part of the ash was sand accidentally present from contact of the smut with the ground while gathering.

The smut was carefully examined for poisonous alkaloids, to see whether the alleged poisonous properties of the smut could be explained by the presence of any organic poison, but not a trace could be detected, although a large quantity (twenty grammes) was used for this purpose.

It is surprising to see the avidity with which cattle will eat the corn-smut, and it seems difficult to explain their appetite for so repulsive a material. Perhaps the presence of sugar (4 per cent.) in the smut may explain this, for the reason that cattle are very fond of sugar.

The conclusion which can be safely drawn from this experiment is, that where cows are gradually brought into the habit of consuming large quantities of smut it does not seem hurtful to them. Whether the same thing would be true where cows, unaccustomed to smut, suddenly gain access to large quantities of it, must remain for future experiment. It is safe to say, however, that any quantity of smut that would be at all likely to exist in a cornfield or on the stalks as fed under normal conditions to the cows of the farmer, would not be dangerous to the health of the animals.

In 1868 Professor John Gamgee, in investigating the "cornstalk disease," fed experimentally forty pounds of corn-smut to two cows, beginning with six and increasing to twelve ounces daily. The smut was fed with ground-grain and chopped-hay. To one cow it was given wet, to the other dry. The cow that received the wet ration gained in weight during the trial, the other lost in weight, but both remained well.

Dr. N. S. Mayo, in discussing the relation of corn-smut to "the cornstalk-disease of cattle," in *Bulletin No.* 58 of the Kansas Experiment Station, records the experience of a farmer living near Manhattan who, believing that corn-smut was liable to produce the disease, took pains to gather the smut from the field. "One night his cattle broke into the enclosure where the smutty corn and smut had been thrown out, and ate all they wished; no injurious effects were noticed."

In *Bulletin No.* 20, United States Department of Agriculture, Bureau of Animal Industry, there is recorded the results of an experiment performed in January, 1894, of feeding corn-smut in large quantities to two heifers. The results are reported as follows:

Beginning on the morning of January 17, 1894, and continuing until noon of February 2 (sixteen and one-half days), the heifers were fed morning and evening with from two to three quarts of a mixture of corn-meal middlings and wheat-bran, and sixteen quarts of smut. The actual quantity of the fungus consumed by one heifer was sixty-one pounds, or a daily average of nearly three and seven-tenths pounds, and by the other sixty-seven and one-half pounds, or a daily average of four and one-fifth pounds. The temperatures of the animals were taken every morning and evening. The animals appeared to be perfectly well throughout the time of feeding, and continued so for several months, during which time they were kept under close observation.

The results of our experiment coincide with those of other experimenters whose records are available, and may be taken as showing that no danger is to be apprehended from the feeding of smutty stalks, either to pregnant dairy-cows or to those in full milk. It is unquestionably true that the feeding of smutty stalks and corn perpetuate the disease from year to year through the medium of the manure. It is otherwise good management to haul manure directly from the stable to the fields, usually in sod, on which the corn is to be planted the following year, although such a course, while economizing the human labor in the care of the cows, brings about the best possible conditions for infecting the corn-crop. No statistics are at hand to show that the corn-smut is more prevalent in recent years than heretofore, although the practice of hauling out manure as fast as it is made has been common for many years.

To prevent the spread of the disease it may be expedient to remove the growing smut-boils before the spores mature, but the expense of going through the cornfield as often as would be necessary to accomplish this purpose would be so great as to render the method out of the question for the ordinary farmer. After the dark-brown masses of spores have become ripe and dry but little advantage can result from cutting them off and leaving them in the cornfield, where they would be blown about by the winds.

---

A New Anæsthetic.—"Orthoform" is the title of a new German anæsthetic, a powdered preparation of benzomethylic ether, which affords immediate relief when applied to deep burns, ulcers, cancers, etc.

Recent researches into the birth of 16,000 foals show that 97 colts are born to every 100 fillies ; that up to the end of the third month of gestation the fœtus is sexless, and that the sex is determined by the bodily condition of the mother in the fourth and fifth months. If the mare and fœtus be well nourished, the sex is likely to be a female.

